# Supplementary material for: H3.3K27M mutant proteins reprogram epigenome by sequestering the PRC2 complex to poised enhancers
Source: eLife. 2018 Jun 22;7:e36696. doi: 10.7554/eLife.36696 (PMC6033537; doi:10.7554/eLife.36696)
Supplement: Supplementary file 1. [file elife-36696-supp1.docx]

**Supplemental Table 1. Cell lines and tissues used.**

| **Cell line/Patient ID** | **Age/Sex** | **Histology** | **Location** | **H3 Status** | **Tissue collection** | **Source** | **Sample Type** |
| --- | --- | --- | --- | --- | --- | --- | --- |
| PED8 | 14/M | DIPG | Pons | H3F3A K27M | Biopsy | Mayo Clinic | Cell line |
| SF7761 | 6/F | DIPG | Pons | H3F3A K27M | Biopsy | UCSF | Cell line |
| SF8628 | 3/F | DIPG | Pons | H3F3A K27M | - | UCSF | Cell line |
| DIPG4 | 7/F | DIPG | Pons | H3.1K27M | Autopsy | Stanford University | Cell line |
| DIPG13 | 6/F | DIPG | Pons | H3F3A K27M | Autopsy | Stanford University | Cell line |
| DIPG17 | 8/M | DIPG | Pons | H3F3A K27M | Autopsy | Stanford University | Cell line |
| KNS42 | 16/M | Malignant glioma |  | H3F3A G34V | Biopsy | Kyushu University | Cell line |
| SF9427 | 9/F | GBM | Frontal | WT | - | UCSF | Cell line |
| MCF7 | 69/F | Breast cancer |  | WT | - | MCF | Cell line |
| MC-2854 (Gliosis tissue) | 13/-** | Gliosis | Right parietal | WT | Biopsy | Mayo Clinic | Tissue |
| MC-5883 (DIPG tissue) | 8/-** | DIPG | Lt/Rt Pons | K27M* | Autopsy | Mayo Clinic | Tissue |

*** Detected by Western Blot, ** Not known**

**Supplemental Table 2. Oligonucleotides used.**

| **Name** | **5’- Sequence -3’** | **Application** |
| --- | --- | --- |
| WT1 sg #1 | ATAAGAGGGGCCGGCGGCGCGGG | Targeting WT1 promoter |
| WT1 sg #2 | GCCAGCTGAGAGCGCGTGTTGGG | Targeting WT1 promoter |
| WT1 ChIP-F | GAGACACCCTCCTCTTCAACC | ChIP-PCR WT1 promoter |
| WT1 ChIP-R | TACCTGAACGGACTCTCCAGT | ChIP-PCR WT1 promoter |
| NGFR ChIP-F | CAGGGAGAAGGTGAAGCCAG | ChIP-PCR NGFR promoter |
| NGFR ChIP-R | TCCCTTAGAGCCTCTCACCC | ChIP-PCR NGFR promoter |
| MME ChIP-F | CCGAAGAGCCCGTGTATTGT | ChIP-PCR MME promoter |
| MME ChIP-R | CCGAAGAGCCCGTGTATTGT | ChIP-PCR MME promoter |
| ACTIN ChIP-F | CCTCATGGCCTTGTCACAC | ChIP-PCR ACTIN promoter |
| ACTIN ChIP-R | GCCCTTTCTCACTGGTTCTCT | ChIP-PCR ACTIN promoter |
| NGFR RT-F | CCTACGGCTACTACCAGGATG | RT PCR |
| NGFR RT-R | CACACGGTGTTCTGCTTGT | RT PCR |
| WT1 RT-F | CACAGCACAGGGTACGAGAG | RT PCR |
| WT1 RT-R | CAAGAGTCGGGGCTACTCCA | RT PCR |
| MME RT-F | AGAAGAAACAGCGATGGACTCC | RT PCR |
| MME RT-R | CATAGAGTGCGATCATTGTCACA | RT PCR |
| ANGPTL4 RT-F | GTCCACCGACCTCCCGTTA | RT PCR |
| ANGPTL4 RT-R | CCTCATGGTCTAGGTGCTTGT | RT PCR |
| PLAGL1 RT-F | CCCAGAAATCTCACCAGTGTG | RT PCR |
| PLAGL1 RT-R | GTGCCTCTTATAGCCCAGCAT | RT PCR |
| GATA3 RT-F | GCCCCTCATTAAGCCCAAG | RT PCR |
| GATA3 RT-R | TTGTGGTGGTCTGACAGTTCG | RT PCR |
| ATF3 RT-F | CCTCTGCGCTGGAATCAGTC | RT PCR |
| ATF3 RT-R | TTCTTTCTCGTCGCCTCTTTTT | RT PCR |
| NDRG4 RT-F | GGCCTCAACCACAAACTATGC | RT PCR |
| NDRG4 RT-R | CCAATCACATACTTGAACCCGAA | RT PCR |
| ZBTB7C RT-F | AGCCAGCCCTACGTCTATGAG | RT PCR |
| ZBTB7C RT-R | CGTTGAGGATGTGCTTGACA | RT PCR |
| BMP4 RT-F | ATGATTCCTGGTAACCGAATGC | RT PCR |
| BMP4 RT-R | CCCCGTCTCAGGTATCAAACT | RT PCR |
| CDKN2A RT-F | GATCCAGGTGGGTAGAAGGTC | RT PCR |
| CDKN2A RT-R | CCCCTGCAAACTTCGTCCT | RT PCR |
| CDKN2B RT-F | GTGAGAGTGGCAGGGTCTG | RT PCR |
| CDKN2B RT-R | TACAGGAGTCTCCGTTGGC | RT PCR |
| *H3F3A* sg | TAGAAATACCTGTAACGATG | SgRNA to cut mouse *H3F3A* gene |
| H3F3A K27M donor DNA | GTACAAAGCAGACTGCCCGCAAA  TCCACCGGTGGTAAAGCACCCAGG  AAACAACTGGCTACAAAAGCCGCT  CGCATGAGTGCGCCCTCTACTGGAG  GGGTGAAGAAACCTCACAGATACA  GGTATTTCTAAAACGTCGAGCAGTG  GGATAG | Donor DNA to insert H3.3K27M mutation in mES cell |
| H3F3A PCR-F | GTGGTAAAGCACCCAGGAAAC | Amplify mouse *H3F3A* locus |
| H3F3A PCR-R | CTATCCCACTGCTCGACGTTTT | Amplify mouse *H3F3A* locus |
| Suz12 RT-F | TTGCAGCTTACGTTACTGGTT | RT PCR |
| Suz12 RT-R | GGAACTTGCCTTATTGGACAACT | RT PCR |
| Enhancer 1-F | ATGGCTCTTGTCTTCACGTTCCT | ChIP-PCR Enhancer 1 |
| Enhancer 1-R | TCCCTGTCCCCATAGTGAGAA | ChIP-PCR Enhancer 1 |
| Enhancer 2-F | GGCCTGGACAAATTCGCATC | ChIP-PCR Enhancer 2 |
| Enhancer 2-R | CCCCTTGACCAGTCAATCCC | ChIP-PCR Enhancer 2 |
| Enhancer 3-F | TTAGTGTGGCACGAAGGACC | ChIP-PCR Enhancer 3 |
| Enhancer 3-R | CCCCCAGTGCATTTCCTCAT | ChIP-PCR Enhancer 3 |
| Actin ChIP-F | TGGGCTAGGCCTTGCTGATGGTAT | ChIP-PCR Actin |
| Actin ChIP-R | TGCAGATGGAGGCAAAGGGT | ChIP-PCR Actin |
| Hoxa1 ChIP-F | TGATTCCTGCCGGCCAATAG | ChIP-PCR Hoxa1 |
| Hoxa1 ChIP-R | GTAGCAGAGTTGCCCCGATT | ChIP-PCR Hoxa1 |
